# Preliminary Analysis of Atmospheric Front-Related VHF Propagation Enhancements for Navigation Aids

**DOI:** 10.3390/s25144455

**Published:** 2025-07-17

**Authors:** Tomasz Aleksander Miś, Wojciech Kazubski, Mikołaj Zieliński

**Affiliations:** 1 Space Research Center of Polish Academy of Sciences, Division of Plasma Physics, ul. Bartycka 18A, 00-716 Warszawa, Poland; 2 Warsaw University of Technology, Institute of Radioelectronics and Multimedia Technology, ul. Nowowiejska 15/19, 00-665 Warszawa, Poland; wojciech.kazubski@pw.edu.pl (W.K.);

**Keywords:** VHF, VOR, propagation, front, internet of drones, navigation

## Abstract

The tropospheric storm fronts were found to cause disruptions in the propagations of VHF (Very High Frequency) radio signals, elevating their signal levels. This is especially important for VHF radio navigation systems, such as VOR (VHF Omnidirectional Range), used for naval, airborne and terrestrial transportation, and as the assisting navigation aids for the smaller vehicles forming the Internet of Drones. This article describes this disruptive phenomenon analytically and shows an experimental verification of the developed formula, presenting the increase in relative VHF signal range by ~1.8 times with decreasing tropospheric refraction. Contrary to popular VHF propagation models, largely averaged and statistics-based, the shown formula can be used simultaneously with meteorological predictions, contributing significantly to the mitigation of radio navigation issues related to stormy weather in the operative range of the Internet of Drones.

## 1. Introduction

**Figure 1:**
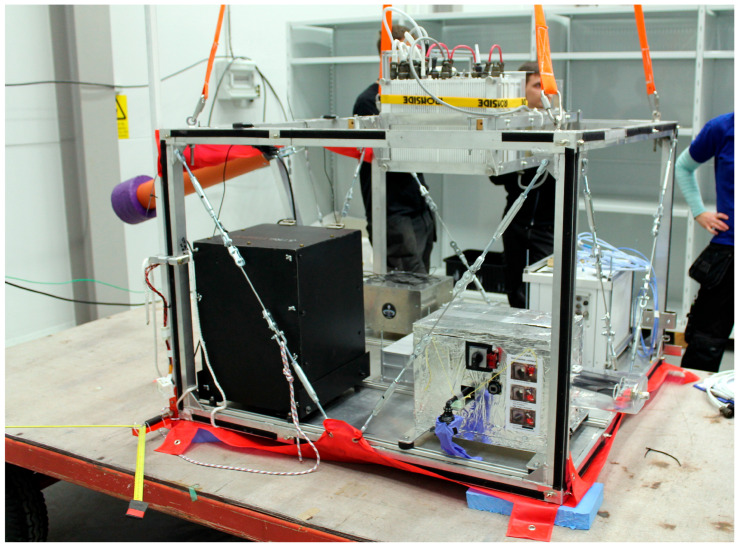
Payload (onboard the M-Egon gondola) of the BX22 stratostat, with yellow VOR VHF navigation antenna (V-type horizontal dipole) system seen mounted on the lower right edge of the frame. Esrange Space Center, 4th October 2016 (Author’s photograph).

VHF radio transmissions (30–300 MHz) have been widely used for communication and navigation purposes over many past decades. Such systems mainly concentrate around the VOR system, widely used for the navigation of aircraft. The system is often assisted with secondary radio navigation aids, such as DME (Distance Measuring Equipment) or TACAN (Tactical Air Navigation System) [1], forming aviation navigation chains effectively covering the air space with acceptable accuracy [2,3,4]. VOR has also been reported as providing viable backup for the GNSS (Global Navigation Satellite Systems), while simultaneously exceeding its typical maximal service altitude towards stratospheric atmospheric layers [5,6] (as seen in Figure 1). In comparison with systems operating on higher frequencies, allowing the employment of wider signal bandwidths, the VHF VOR system still requires much more moderate transmitting equipment to cover similar or greater areas [7], offering the possibility for reliable and inexpensive navigation aid to airborne, naval and terrestrial transportation.

As the market of unmanned vehicles is expanding rapidly into airborne, naval and terrestrial domains, the creation of a networked system called the Internet of Drones (IoD) has created a new large recipient for reliable and easily accessible navigation, not solely relying on the GNSS systems [8]. IoD may include small airborne delivery drones, adding to the global logistic network [9] or mobile communication repeaters for naval navigation [7]. Recent progress in Ukrainian drone industry development showed its importance and effectiveness in modern remotely controlled warfare [10,11], which requires a sufficient level of resilience towards the potential interference to the GNSS. In civilian application, a worldwide well-adopted IoD is formed, e.g., by semi-autonomous mobile delivery robots, navigating city streets primarily using GNSS [12]. Wide accessibility of VOR, its cooperation with GNSS and wide availability of commercial off-the-shelf receivers allow it to be easily employed in the main and/or redundant IoD navigation systems; this is already included in the discussion of the dedicated communication protocols [8].

VHF radio propagation can be estimated using numerous prediction models, taking into account statistical data on the signal coverage over terrain with given features, during different seasons of the year and changing weather. Such models include (but are not limited to) the methods of Longley–Rice [13], Okamura–Hata [14], ITU P.1546 [15] with ameliorations [16], ITM—Irregular Terrain Model [17], Kazantsev [18] or SEAMCAT [17]. These models, however, present an average-based approach to the definition of the propagation path, which is unable to accurately predict in short time environmentally related changes caused by detected ephemeral/short-lived external factors, e.g., heavy precipitations, storms or sporadic ionospheric layers, which would require a more specific analytical approach and more detailed specific data instead of an averaged range of simplified parameters. The latter issue (sporadic ionospheric layers) has been investigated to influence particularly aircraft navigation systems and FM-CCIR broadcasting [19], resulting in anomalous long-distance propagations [20], intense fluctuation of the ground-received signal strength [21] and, as a sort of side effect, possibilities to investigate the 2D structure of the E sporadic ionospheric layer [22].

The influence of the storms on the VHF propagation—and, in effect, the VOR navigation system’s coverage—manifests itself in a front-related elevation of the signal strength due to a near-step change in the temperature, affecting directly the tropospheric refractive index to which the VHF range is sensitive. Such changes would be able to affect the stability of the VOR reception, especially on low/ground altitudes (signal strength pulsations), as well as enable the receptions of ephemerides—signals from VOR navigation chains from very distant locations, disrupting the overall navigation process. Storms, however, are effectively (and with sufficient time advance) predicted by various weather services; this would also enable the prediction of the propagation changes for given VOR stations, increasing the system’s resistance to service delivery failure.

In this article, the storm front-caused propagation change for the VHF range is explained analytically. The developed formulas are then tested on an exemplary data set from VOR stations monitoring in Central Poland, showing the simplicity of their application and non-negligible effect the storm fronts have on the VHF transmissions.

## 2. Theoretical Approach to Front’s Influence

As the typical repetitive storm front structure involves a near-step change in temperature in the zone where two different air masses collide [23], the tropospheric refractive index *n* [−] would be the main factor analyzed in the front’s influence on the VHF propagation. It is, however, more convenient, for practical reasons, to operate with the refractive indicator *N* [−], which is defined as follows [18,24]:

(1) $$N=10^{6}(n-1)$$

**Figure 2:**
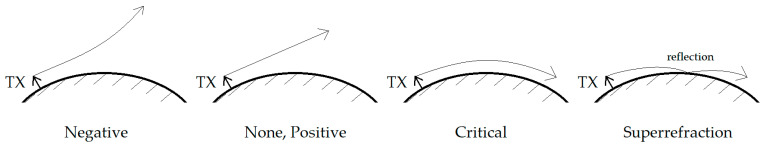
Visual representation of the shapes of the trajectory types (arrows) for different types of refraction from Table 1 (around a spherical Earth surface).

The first derivative in the vertical altitude domain *H* of the refractive indicator *N*—*dN*/*dH*—can be used as a tool defining the tendency of the actual propagating radio wave trajectory. Table 1 and Figure 2 present the crucial values and value ranges for these trajectories [18,25].

The refractive indicator *N* can be calculated using the Essen–Froom formula [18,25]:

(2) $$N=\frac{77.6}{T}\left(p+\frac{4810{\cdot e}_{air}}{T}\right)$$

where *T* is the air temperature [K], *p* is the air pressure [Pa] and *e_air_* is the water vape pressure—for this analysis, in tropospheric conditions without substantial temperature differences—approximated to the water content of the clouds formed at the boundary of two air masses [kg/m^3^]. According to Nikandrov [26], for the Nimbostratus storm cloud, the water content is expected to be between 6 × 10^−4^ and 13 × 10^−4^ kg/m^3^ (mean value: 9.5 × 10^−4^ kg/m^3^).

**Figure 3:**
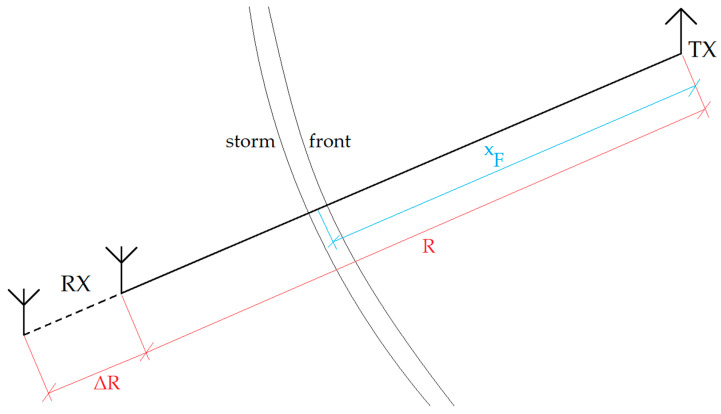
The schematic of the VHF/VOR propagation through a storm front.

**Figure 4:**
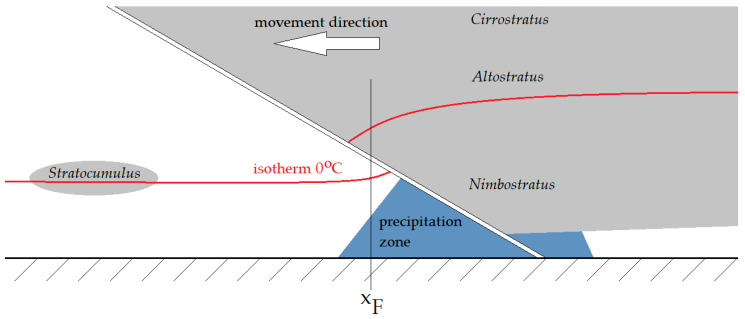
The general profile view of the storm front and its course of the 0 °C isotherm.

From Formula (2), it can be seen that the tropospheric refraction of the propagation path for VHF is mainly the function of the environment’s temperature. Its near-step change appears at the contact of two different air masses forming the storm front—in Figure 1 at the distance *x_F_* from the VHF/VOR transmitter TX. On the receiving end RX, the change in the effective range of the signal Δ*R*, directly resulting from the increase in the signal strength due to the change in *N* with *T*, is expected. The Δ*R* range change is schematically depicted in Figure 3. Figure 4 presents the general structure of the storm front and the course of the 0 °C isotherm (based on general aviation approach, as in [23]).

**Figure 5:**
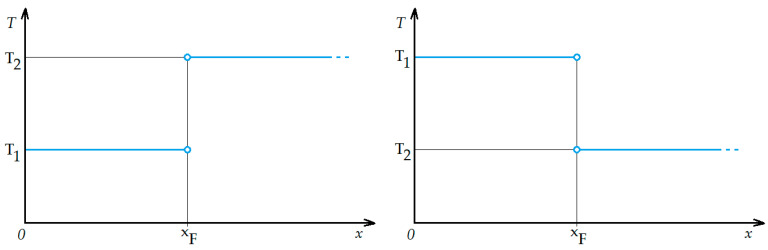
The sigmoid function cases described by Formula (4), approximating the non-continuous change (blue lines) in the 0 °C isotherm depicted in Figure 4 for both possible cases (for different movement directions of the storm front).

The near-step change in the temperature in the storm front can be approximated by a variety of complex mathematical expressions with numerous unknowns [27], many of which do not maintain continuity in the distance (*x* [m]) domain, posing difficulties in calculation. However, an approximation of this change could be expressed with a sigmoid function *H*(*x*) having the following form [28]:

(3) $$H\left(x\right)=\frac{1}{1+e^{-2kx}}$$

for *x* > 0, and *k* [−] is an arbitrarily chosen constant, refining the shape of the sigmoid (the greater the value of *k* > 0, the stronger the sigmoid’s resemblance to the Heaviside function). An important assumption in the description of the storm front’s temperature-related refractive indicator discontinuity shall be the vertical temperature change *T*(*x*) from *T*_1_ to *T*_2_ appearing at the position *x_F_* (Figure 1)—this, using Formula (3), this can be expressed as

(4) $$T\left(x\right)=T_{1}+\frac{T_{2}-T_{1}}{1+e^{\pm 2k\left(x-x_{F}\right)}}$$

with the interchanging signs at the exponential function’s exponent indicating two analytical cases: *T*_1_ > *T*_2_ or *T*_1_ < *T*_2_ (both happening at *x_F_*), which depend on the direction of the movement of the storm front. Figure 5 presents both possibilities for this function—the choice of the actual case depends on the orientation of the front, determining the shape of the 0 °C isotherm.

At the later stage of the calculations, the lower temperature value can be attributed to the 0 °C isotherm, simplifying the approach. This expression can be implemented in the Formula (2), yielding

(5) $$N\left(x\right)=77.6\left(\varrho R_{air}+\frac{4810{\cdot e}_{air}}{{\left[T_{1}+\frac{T_{2}-T_{1}}{1+e^{\pm 2k\left(x-x_{F}\right)}}\right]}^{2}}\right)$$

where (according to Clapeyron’s equation) ϱ is the air density [kg/m^3^] and *R_air_* is the air’s gas constant [J/mol × K]. The first derivative in the distance domain of *N*(*x*) shall therefore be equal to

(6) $$\frac{dN}{dx}=\pm 1493024\frac{e_{air}k\left(T_{1}-T_{2}\right)e^{2k\left(x-x_{F}\right)}\left(e^{2k\left(x-x_{F}\right)}+1\right)}{{\left[T_{1,2}e^{2k\left(x-x_{F}\right)}+T_{2,1}\right]}^{3}}$$

Substituting specific values for *k* and *x* yields

(7) dNdx|x=xFk=20 =−59720960⋅eairΔTT0

where Δ*T* is the temperature change at the storm front [K] and *T*_0_ is the temperature on the 0 °C isotherm: 273.15 K. As Δ*T* is the temperature change in the vertical direction, this derivative shall describe the vertical change in the refractive indicator *N*, which remains compliant with the Table 1 data and descriptions. It is also worth noting that this expression does not finally depend on the exact value of the *x_F_*—the required parameters are solely the cloud’s water content and the near-step temperature change at the storm front.

According to Schmidt [23], the approximate altitude change in a warm storm front is 2000 m for the positioning of the 0 °C isotherm. This can be recalculated into temperature change at the warm storm front using a tropospheric formula [29]:

(8) $$T\left[℃\right]=15.04-0.00649\cdot H[m]$$

where *H* [m] is the altitude change. For the mentioned 2000 m, this temperature change shall be equal to 13 °C, by definition identical to 13 K.

**Figure 6:**
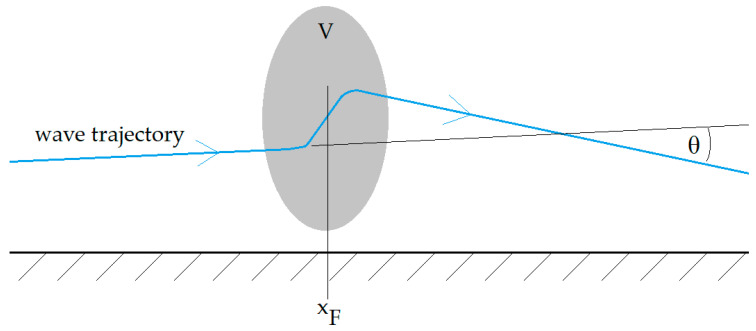
The storm front 0 °C isotherm course modeled as a dispersion zone of the volume *V* (gray zone) of the troposphere (altitude below 12,000 m), valid for frequencies above 3 MHz. Physically, in typical applications, volume *V* usually consists of localized temperature- and density-related atmospheric heterogeneities which affect the radio wave trajectory. In the approach in this article, this heterogeneity is assumed to be the temperature drop related to the storm front.

From experimental measurements’ point of view, the recorded parameter is the VHF signal strength in dBm. If the analyzed front-related phenomena are treated as an exceptional case of a tropospheric dispersion, the formula combining the power at the receiving end of the propagation path (RX in Figure 1) with the range *R* can be expressed as [18]

(9) $$P_{RX}=\frac{P_{\Sigma }G_{1}G_{2}\lambda^{2}}{4\pi^{3}R^{4}}V\sigma (\theta )$$

where *P*_Σ_ is the radiated power (at the TX), *G*_1_ and *G*_2_ are the antenna gains at the transmitting and receiving locations, *λ* is the signal’s wavelength [m], *R* is the obtained range [m], *σ*(*θ*) is the area of the tropospheric dispersion surface, with *θ* being the angle between the propagation trajectories that enter and exit the dispersion zone, and *V* is the volume of the troposphere in which the dispersion appears. The schematic of the dispersion region in this approach is shown in Figure 6.

The volumetric parameter *V* can be defined as [18]

(10) $$V=\frac{1}{8}aR^{2}\alpha_{V}\alpha_{H}$$

where *a* is the Earth’s radius and *α_V_* and *α_H_* are the half-radiated-power angles in vertical and horizontal planes, respectively.

The function *σ*(*θ*) has the formula incorporating the actual and averaged over a given time values of the altitude derivative of the refractive indicator *dN*/*dH* [18]:

(11) $$\sigma \left(\theta \right)≅\frac{4\pi^{2}\lambda }{\theta^{5}}\bar{{\left(\frac{dN}{dH}-\bar{\frac{dN}{dH}}\right)}^{2}}$$

The employment of (9)–(11) is reserved exclusively for the VHF range. As in Figure 1, the storm front is predicted to change the obtained range *R* (no storm front—index ‘1’) by Δ*R* (with storm front—index ‘2’), which can be included in the power Formula (9):

(12) $$P_{RX2}\left(R_{2}\right)=P_{RX2}(R+\Delta R)$$

The ratio of these two cases can be therefore written as

(13) $$\frac{P_{RX2}}{P_{RX1}}=\frac{R^{2}}{{\left(R+\Delta R\right)}^{2}}\frac{{\bar{{\left(\frac{dN}{dH}-\bar{\frac{dN}{dH}}\right)}^{2}}}_{2}}{{\bar{{\left(\frac{dN}{dH}-\bar{\frac{dN}{dH}}\right)}^{2}}}_{1}}$$

This expression can be simplified by taking into account the actual values of the altitude derivatives of the refractive indicators only:

(14) $$\frac{P_{RX2}}{P_{RX1}}=\frac{R^{2}}{{\left(R+\Delta R\right)}^{2}}\frac{{\left(\frac{dN}{dx}\right)}_{2}^{2}\;}{{\left(\frac{dN}{dH}\right)}_{1}^{2}}$$

The change in the obtained signal range due to the storm front activity is therefore equal to

(15) $$\Delta R=R\left({\left(\frac{P_{RX2}}{P_{RX1}}\cdot \frac{{\left(\frac{dN}{dH}\right)}_{1}^{2}\;}{{\left(\frac{dN}{dx}\right)}_{2}^{2}}\right)}^{-\frac{1}{2}}-1\right)$$

with the storm front (‘2’) derivative expressed by Equation (7) and the no-front derivative (‘1’) defined from external/weather-related sources. Naturally, the power measured in dBm at the receiving point needs to be recalculated to comply with the linear characteristic of the power ratio included in (15). This formula also inherits—from (9)–(11)—the assumption of being used for VHF exclusively.

## 3. Experimental Application

**Figure 7:**
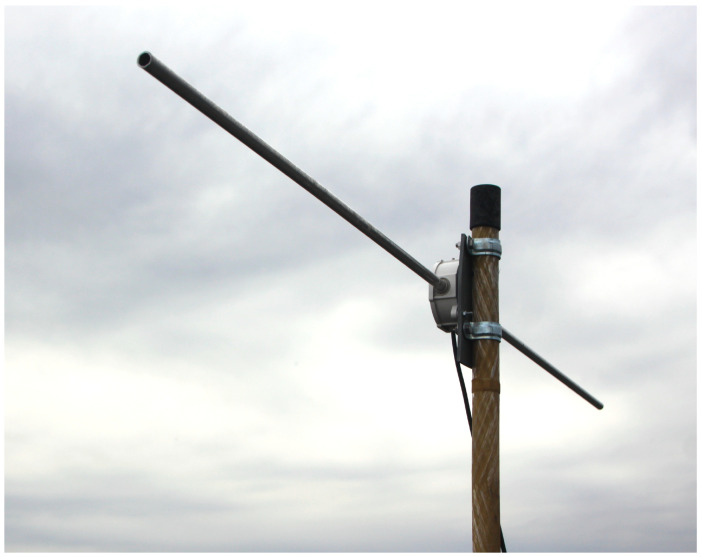
The receiving horizontal dipole antenna (same polarization as in Figure 1). The total length of the dipole is twice 0.6625 m.

**Figure 8:**
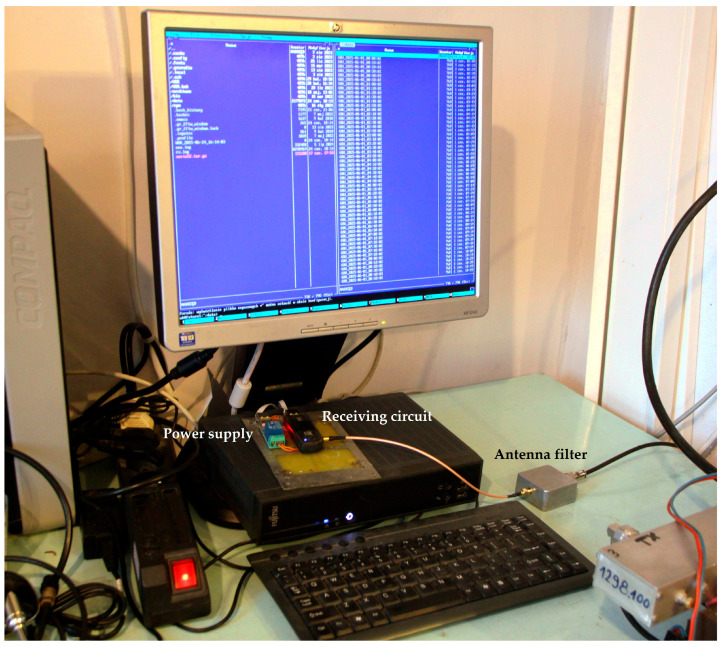
The receiving circuit with shown data logging on a PC.

**Figure 9:**
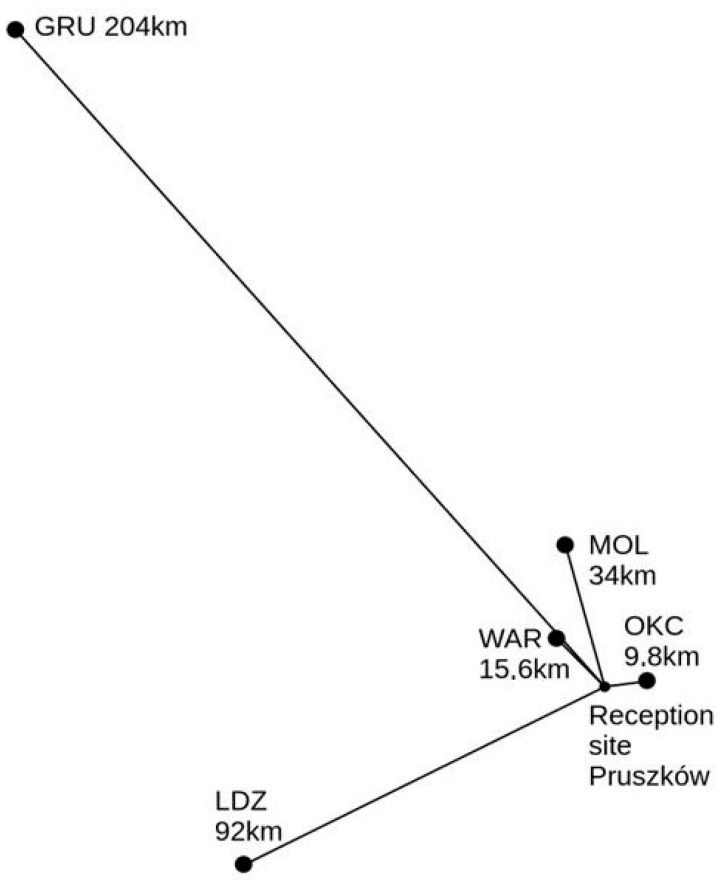
The locations and distances from the transmitting VOR stations, described by their callsigns, to the receiving station in Pruszków, Poland.

**Figure 10:**
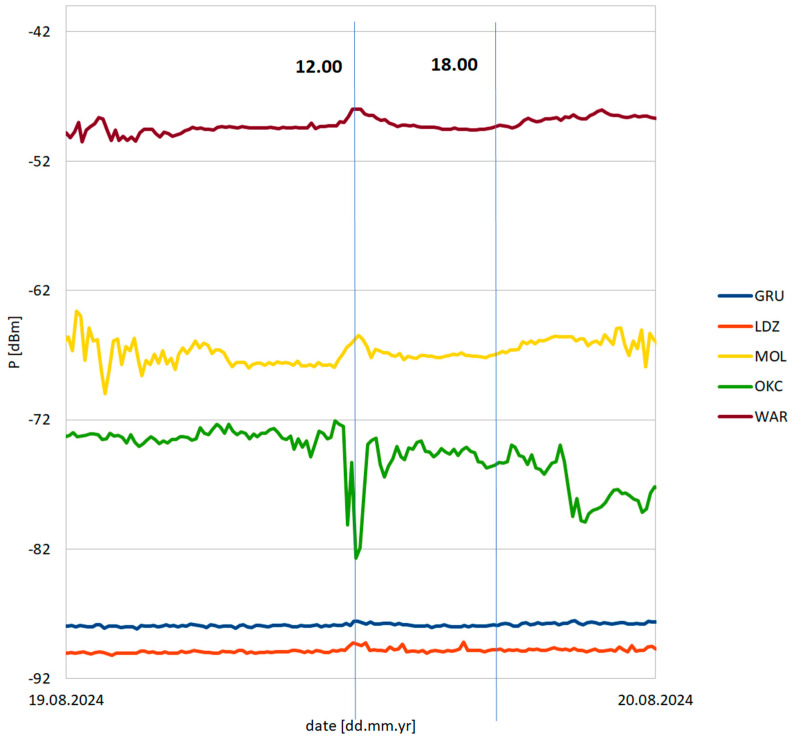
Measured VOR stations’ signal strengths between 19th and 20th August 2024. The vertical lines indicate the time window between which the storm had appeared.

The developed Formula (15) has been verified using VOR signal data monitored in August 2024 from a station in Pruszków near Warsaw, Poland (52.1585° N 20.8177° E). Figure 7 and Figure 8 present the receiving antenna system, affixed to the roof of a residential building, and the receiving circuit with data logging (custom software, Warsaw University of Technology, Poland) on a PC computer. The antenna—half-wavelength dipole for 113 MHz center frequency—is symmetrized by ferrite cores on the feeder line. The details of the monitored VOR stations are shown in Figure 9 and in Table 2. It was identified that in the considered period, two air masses collided, resulting in a later propagating storm—the basic assumption required to validate the formula. Figure 10 presents the registered signal strengths for five VOR stations for the period covering the air masses collision around 12.00 PM local time. The phenomena briefly appeared in the north-eastern direction, visibly affecting the propagations of three VOR stations: LDZ, MOL and WAR. The OKC station changed its transmission mode at that moment, and the GRU station showed negligible influence. In the latter case, the absence of changes can be explained by greatest path attenuation and the fact that the storm region covered only a small fraction of the path. Changes in these signal strengths are shown collectively in Table 3.

**Figure 11:**
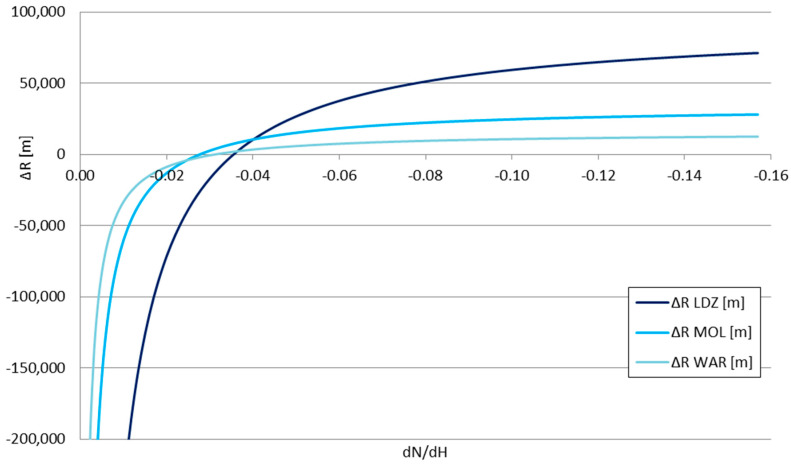
The change in the obtained signal range Δ*R* as a function of the derivative of the tropospheric refraction indicator *dN*/*dH*.

**Figure 12:**
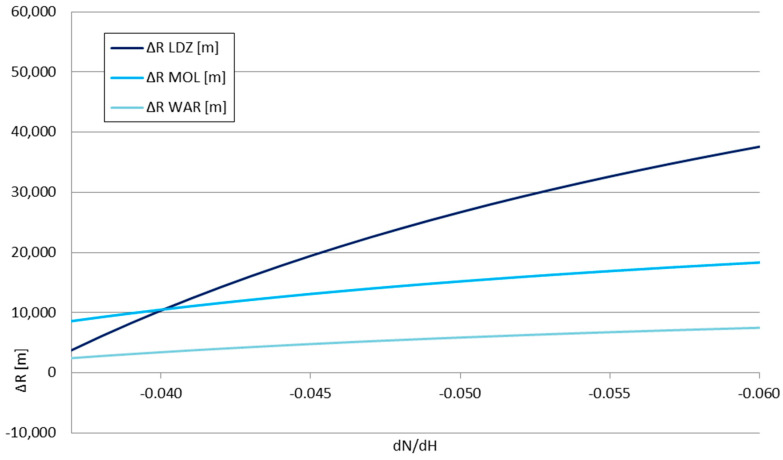
The change in the obtained signal range Δ*R* as a function of the derivative of the tropospheric refraction indicator *dN*/*dH—*enlarged part around the limiting value of −0.04 (positive refraction).

**Figure 13:**
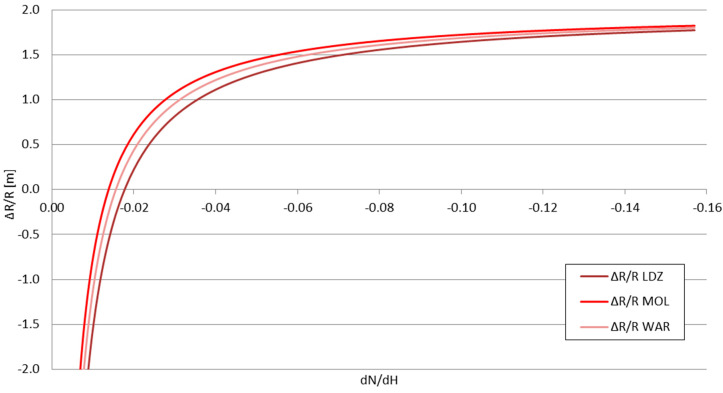
The relative change in the obtained signal range Δ*R/R* as a function of the derivative of the tropospheric refraction indicator *dN*/*dH*.

**Figure 14:**
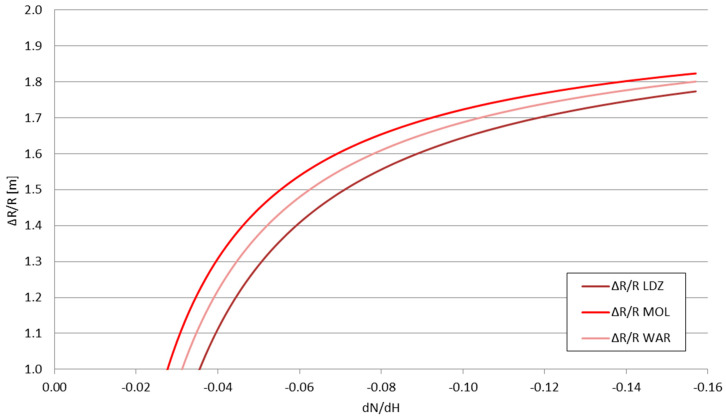
The relative change in the obtained signal range Δ*R/R* as a function of the derivative of the tropospheric refraction indicator *dN*/*dH—*enlarged part around the limiting value of −0.04 (positive refraction).

The presented values form the inputs for the *P_RX_*_1_ and *P_RX_*_2_ in Formula (15). The *dN*/*dx*|_2_ value—the derivative of the refraction indicator in storm front conditions—taking into account the previously mentioned temperature data, is equal to −0.03619—classified as positive weak, according to Table 1 descriptions. The refraction indicator of the troposphere before/around the storm front can be treated as a variable, allowing the calculation of the change in the obtained signal range Δ*R*—shown in Figure 11 and Figure 12—and the relative range change Δ*R/R* for each considered station—shown in Figure 13 and Figure 14.

## 4. Discussion

The obtained Δ*R* and Δ*R*/*R* values for the signal changes shown in Table 3 strictly align with the refraction types outlined in Table 1. For the negative refraction range (*dN*/*dH* between 0 and −0.04), the analyzed storm front effect is the opposite—it is able to reduce the achievable VOR signal range; for the compared stations, the weakest station (LDZ) is more vulnerable to this effect than the strongest one (WAR). For the positive refraction, however, the weakest station is more susceptible to the elevation of the achieved range than the stronger stations—the gained range for the weaker station makes it more comparable to the stronger stations without the influence of the storm front. This directly indicated the storm front’s possibility of favoring the appearance of ephemerides in the navigation signals.

The influence of the storm front, when measured by the Δ*R*/*R* values, remains largely similar to all stations, regardless of their signal strengths, with minor differences between the stations (stronger signals rated slightly higher than the weaker signals). All values for the positive refraction range tend to converge to the approximate value of 1.8, with the largest differences between them noted for the positive normal and early-positive strong refractions. For the negative refractions, however, the signals’ achieved range may drop by up to two times.

Naturally, the actual change in Δ*R* and Δ*R*/*R* depends on the actual refraction type before the arrival of the storm front, which is to be defined from external meteorological observations.

**Figure 15:**
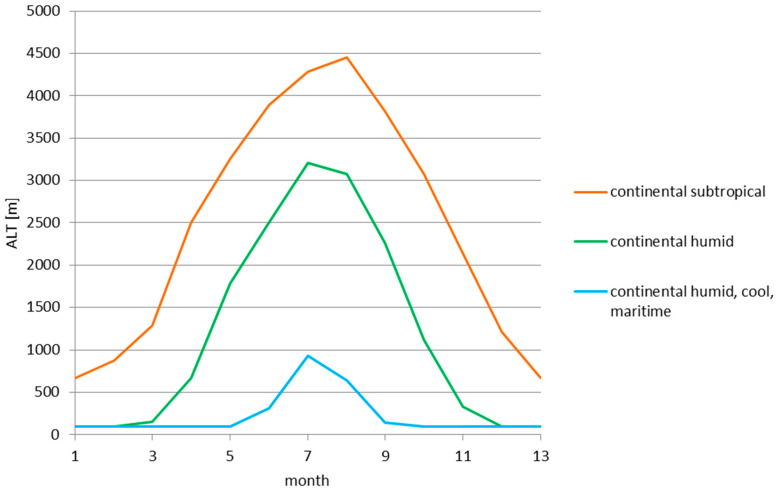
The altitude change in the 0 °C isotherm for the subsequent months (averaged per month) for different climate zones, based on archive data from Nikandrov [26].

An important future consideration in the application of the described method is the seasonal change in the 0 °C isotherm altitude, depending heavily on the local climate conditions. Figure 15 presents the seasonal change in this altitude for subsequent months, based on archive data from Nikandrov [26]. All three curves shown belong to the continental type of climate, but with different characteristics. The significant relative change in the 0 °C isotherm altitude (reaching nearly 4 km for the continental subtropical climate throughout the year) indicates also the significant change (in the scale of the year) in the altitude of the described phenomenon affecting the VHF propagation through a storm front. As the storm front causes the non-continuous temperature drop, as presented in Figure 4, the altitude of this drop—referred to as the 0 °C isotherm—shall also differ. For the VHF propagation over large distances and uneven terrain (mountains, high vegetation, buildings), with the increasing size of the drop (the difference between *T*_1_ and *T*_2_), larger range improvements Δ*R* would be expected for the summer months and for the subtropical and humid continental climates than for the winter months and the cool, maritime continental climate due to the higher occurrence altitude of the 0 °C isotherm. The exact scale of influence of this yearly variation remains an open question for future research, but remains a potentially important factor in VHF communication planning in climate zones where the summer and winter temperature conditions differ significantly.

## 5. Conclusions

This article proposed a new method of assessing the influence of a tropospheric storm front on the propagation of the VHF radio signals, especially meant for the navigation and the Internet of Drones (IoD). The analytical formula, based on the actual parameters of the environmental condition instead of typically employed statistical models, was verified experimentally using the IoD-applicable VOR navigation system stations in Poland, showing clear influences on the achieved signal ranges.

The differences in propagation introduced by such weather events are significant enough to be considered as a parameter that is neither entirely nor precisely covered by the propagation models relying on statistical models and averaged environmental parameters. As many effective methods of storm front tracking and analysis in real time are already in use [30], the presented method can be used simultaneously with weather services, adding to the real-time prediction of the radio navigation signal coverages, by assessing of the signal strength’s change if a storm front is detected forming and/or travelling on the monitored propagation path. This is especially important due to the relatively easy tracking of the storm fronts and their predicted more frequent appearances [31]. The fact that their influence is largely local [32] and stronger at the surface of the ground [33], radio-navigated mobile units—naval, airborne and land drones/robots—are especially vulnerable to navigation disruptions caused by these events (similarly to the effects caused by sporadic ionospheric layers—signal ephemerides, causing errors in station signals’ decoding); with the presented method, it would be possible to predict unfavorable VHF navigation propagation situations, monitor them in real time and include analytically precise precaution measures as soon as it is possible.

## Figures and Tables

**Table 1:** Characteristics of various types of tropospheric refraction types.

Refraction Type	*dN*/*dH*	Trajectory Type
Negative	>0	Upward-directed
None	0	Ground-parallel
Positive weak	(0; −0.04)	Approximately ground-parallel
Positive normal	−0.04	Approximately ground-parallel
Positive strong	(−0.04; −0.157)	Approximately ground-parallel
Critical	−0.157	Following the curvature of the ground
Supercritical	<−0.157	Off-ground reflections appearing

**Table 2:** Received VOR stations’ details.

Station Callsign (Name)	WGS−84 Coordinates	Transmitting Frequency
OKC (Okęcie)	52.1697° N 20.9600° E	113.45 MHz
MOL (Modlin)	52.4525° N 20.6778° E	116.6 MHz
WAR (Zaborówek)	52.2592° N 20.6572° E	114.9 MHz
GRU (Grudziądz)	53.5211° N 18.7814° E	114.6 MHz
LDZ (Łódź)	51°46′34″ N 9°37′29″ E	112.4 MHz

**Table 3:** Changes in signal strengths of selected VOR stations.

Station Callsign	Signal Strength—No Storm Front	Signal Strength—with Storm Front
LDZ	−89.51 dBm	−89.34 dBm
MOL	−67.79 dBm	−65.45 dBm
WAR	−49.27 dBm	−47.98 dBm

## Data Availability

VOR propagation data is available upon request: wojciech.kazubski@pw.edu.pl.
